# Tensile Deformation Behavior of Typical Porous Laminate Structure at Different Temperatures

**DOI:** 10.3390/ma13235369

**Published:** 2020-11-26

**Authors:** Ping Wang, Ye-Da Lian, Zhi-Xun Wen

**Affiliations:** School of Mechanics, Civil Engineering and Architecture, Northwestern Polytechnical University, Xi’an 710072, China; wangpingping@mail.nwpu.edu.cn (P.W.); zxwen@nwpu.edu.cn (Z.-X.W.)

**Keywords:** digital image correlation technology, Ni-Cr-W superalloy, microstructure, fracture mechanism

## Abstract

In this study, the Ni-Cr-W superalloy GH3230 is used as the test material. According to the actual structure of the flame tube, a porous laminate structure specimen is designed. The structure consists of impact holes, overflow holes and pin fins. High-temperature tensile tests at 650 °C, 750 °C and 850 °C were carried out to study the high-temperature mechanical properties and fracture mechanism of the specimens of porous laminate structure, and the strain nephogram of the specimens were obtained by digital image correlation (DIC) technique. Due to the large number and dense arrangement of overflow holes, an obvious hole interference effect can be found from the strain nephogram. The stress concentration around the pore and the interference between the pores provide priority places and paths for the initiation and propagation of microcracks. The test found that the microcracks of the porous laminate structure first occurred around the hole, the overflow surface fractured first, after which the impact surface fractured. The strength of the alloy exhibits a significant temperature sensitivity to temperature. From 650 °C to 750 °C, the ultimate strength (σ_b_) and yield strength (σ_0.2_) decrease slightly, but they decrease significantly at 850 °C. The microstructure of the fracture surface shows that all microcracks occur at the interface between the matrix and the carbides but that the fracture mode of the specimens gradually changes from intergranular fracture to transgranular fracture as the temperature increases. Due to the pinning effect of the intracrystalline diffusive solute atoms on the dislocations, the stress-strain curves of the high-temperature tensile tests at 650 °C and 750 °C showed zigzag characteristic fluctuations during the strengthening stage.

## 1. Introduction

In general, in order to make superalloys suitable for long-term use under critical temperature and stress conditions, a large number of alloying elements such as chromium, molybdenum, boron and C are added to the alloys [1,2]. Among them, the Ni-Cr-W superalloy is a kind of nickel based deformation superalloy with solid solution strengthening and carbide dispersion strengthening. A large amount of refractory elements such as W and Cr are added to the alloy to improve the strength of the matrix, and a small amount of C element is added to form dispersed carbides to hinder the grain growth and strengthen the grain boundary [3,4,5]. Therefore, it has excellent fatigue creep mechanical properties, corrosion resistance and oxidation resistance, and is widely used in gas turbine and aero-engine components, such as blades, flame tubes, etc. [6,7,8,9].

At present, some research has been done on the microstructure and mechanical properties of Ni-Cr-W superalloys. Han y et al. [10] studied the effect of carbides formed by grain boundary element segregation on the mechanical properties and microstructure of Ni-Cr-W superalloys. It is found that, with the increase of the heat exposure time, the morphology of M_6_C carbide changes from grain to strip, which reduces the mechanical properties of the material. Hu and Tang et al. [11,12] studied the precipitation behavior of M_23_C_6_-type carbides at the grain boundary of a Ni-Cr-W superalloy and their effect on the mechanical properties. The results show that the large interfacial energy at the large angle grain boundary is conducive to the formation of M_23_C_6_-type carbides and that the decrease of the tensile strength and yield strength is mainly caused by the fracture of the M_23_C_6_-type carbide. Hao et al. [13] studied the effect of the C element content on the microstructure and fracture properties of Ni-Cr-W-Fe superalloys and found that a high C element content was prone to forming M_23_C_6_ carbides, which accelerated the crack along the grain boundary extension. MQ et al. [14] studied the effect of grain refinement on the microstructure and mechanical properties of Ni-Cr-W superalloys. Bai et al. [15] studied the effect of temperature on the tensile mechanical properties of Ni-Cr-W superalloys. In summary, although there have been some studies on the mechanical properties of this type of superalloy, most research work has not considered the impact of the real structure in engineering practice on the material properties, especially the high-temperature mechanical properties. With the increase of the turbine inlet temperature and the increase of engine efficiency, higher requirements are put on the operating temperature of hot components such as blades and combustion chambers. At present, film-cooling hole cooling methods are mostly used. However, the existence of dense vent membrane holes makes the structural stress state complex, and cracks are more likely to occur around the holes [16,17]. Therefore, it is necessary to understand the mechanical properties of the film-cooling holes’ structure.

In this paper, according to the actual structure of the combustion chamber, the porous laminate structure was designed with a Ni-Cr-W superalloy GH3230, and its high temperature tensile mechanical properties were studied at different temperatures. The surface strain field of the structure was obtained by Digital image correlation (DIC) technology.

## 2. Experimental Material and Process

### 2.1. Material and Specimens

**Figure 1:**
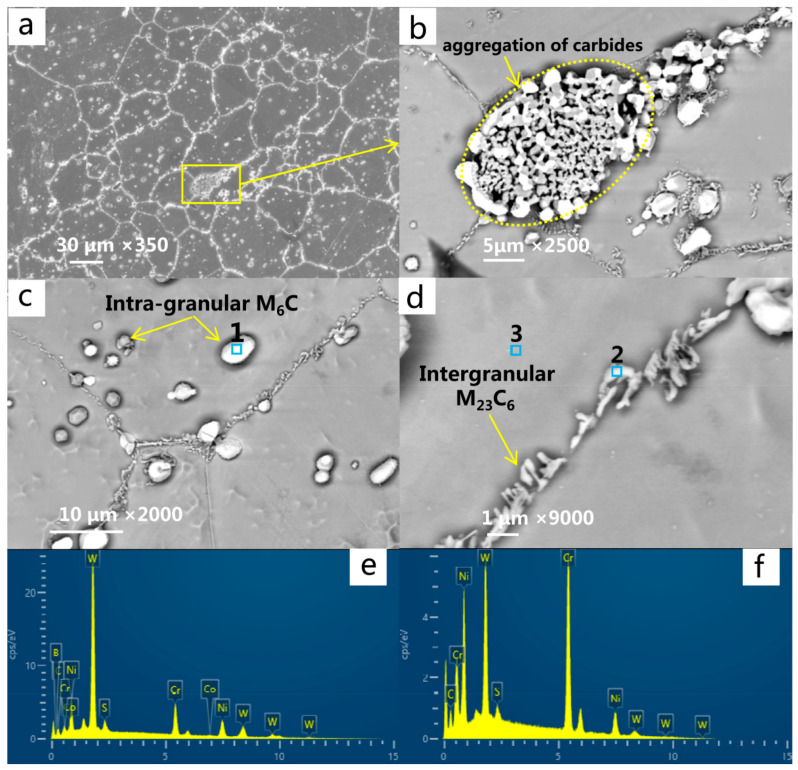
Scanning electron microscope (SEM) images of the microstructure of the GH3230 superalloy: (**a**) microstructure and morphology; (**b**) segregation and aggregation of intergranular carbides; (**c**) morphology and distribution of M_6_C-type carbides in grains; (**d**) morphology and distribution of M_23_C_6_ type between the grains; (**e**,**f**) the results of the EDS analysis at positions 1 and 2 in the figure, respectively.

The deformed superalloy GH3230 was used in the present experiment. The experimental GH3230 superalloy materials are all from Beijing Institute of Aerial Materials (Beijing, China). The nominal chemical composition is given in Table 1. The ingot was prepared by vacuum induction furnace + electroslag remelting, and then the microstructure was homogenized by standard heat treatment (1310 °C/1 h + 1320 °C/2 h + 1330 °C/2 h + 1340 °C/4 h, 1120 °C/4 h (AC), 870 °C/32 h (AC) (AC: air cooling)). Finally, the original material was prepared by a cold rolling process. The test alloy is a Ni-Cr-based solid solution strengthened deformation superalloy, which is an isotropic polycrystalline material. After being grinded, polished and electrochemically etched in a solution of 95 mL HCL + 10 mL CH_3_COOH (by using a voltage of 4 V and corrosion time of 10 s), the grain distribution and microstructure of the specimen were observed through a scanning electron microscope (SEM), and the results are shown in Figure 1. The microstructure of the alloy is mainly composed of γ matrix, M_6_C carbide and M_23_C_6_ carbide (Figure 1a). Granular M_6_C carbide is the primary carbide, which is uniformly distributed in the alloy matrix (Figure 1c). The microstructure of M_23_C_6_ carbide is linear or lamellar, mainly distributing at the grain boundary (Figure 1d). The carbide is enriched in some regions of the grain boundary (Figure 1b). According to the results of the EDS analysis, M_6_C carbide mainly contains Ni, W, Co, W, etc., while M_23_C_6_ type carbide is mainly enriched in Ni, W, Cr, etc., as shown in Figure 1e,f.

**Figure 2:**
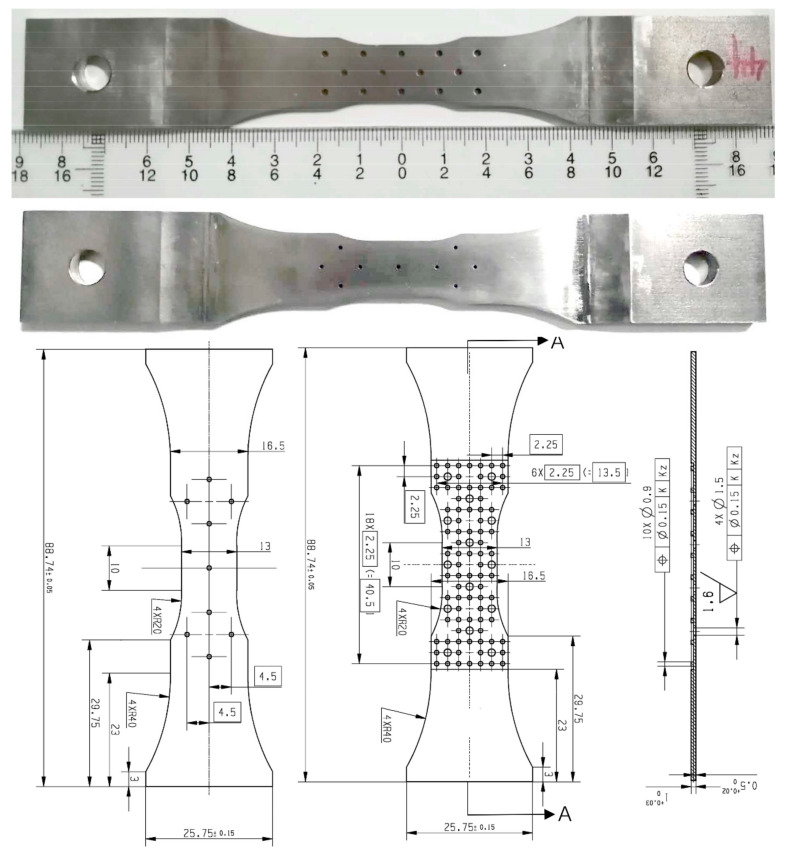
Physical picture and geometry schematic of the specimens (unit: mm).

In order to simulate the structure of the flame tube in actual engineering and in combination with its structural characteristics, a double-layer thin wall plate structure is designed. The structure is composed of pin fins and two kinds of film-cooling holes: overflow holes and impact holes. The number and arrangement of the two kinds of holes are different. In order to eliminate the influence of the recast layer on crack initiation around the holes, picosecond laser drilling technology is used for punching. The diameter of the overflow hole is 1.5 mm, the arrangement is a triangle symmetry, and the plate thickness is 0.5 mm. The hole diameter of the impact hole is 0.9 mm, the arrangement mode is a rhombic distribution, and the plate thickness is 0.8 mm. The pin fin connecting the two layers is a cylinder with a diameter of 0.15 mm and a height of 0.25 mm. The actual picture and detailed size of the specimen are shown in Figure 2.

### 2.2. Experimental Procedure

**Figure 3:**
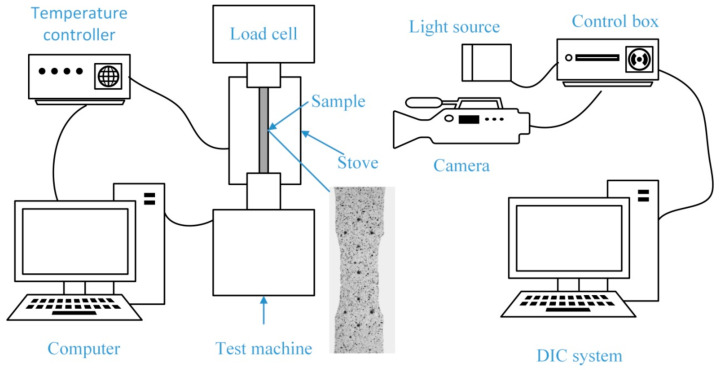
Schematic diagram of the test process and preformed speckle pattern on the surface of the specimens.

In order to study the mechanical behavior and microstructure evolution of porous laminates under a high-temperature, uniaxial tensile condition, the experiments at 650 °C, 750 °C and 850 °C were carried out on a high-temperature electronic creep testing machine (RDL100, Changchun Machinery Experimental Equipment Co., Ltd., Changchun, China) according to the actual working conditions, and each condition was tested twice. The test process is controlled by displacement, and the tensile rate is 0.2 mm/min. During the test, a K-type thermocouple was attached to the upper, middle and lower sections of the specimen to monitor the temperature in real time. In order to study the interference effect between film-cooling holes and the thermal strength of the structure, digital image correlation (DIC) (XTDIC Version 8.2.1, Xi’an Jiaotong University, Xi’an, China) was used to measure the strain field on the surface of the specimen. Before the test, high-temperature paint was sprayed on the surface of the test piece to make the speckle area. During the whole experiment, a high-temperature lens was used to collect images every 10 s. The images are processed by the DIC system, and the changes of the gray value in the speckle region are calculated. The strain data of the specimen surface is obtained, and the strain nephogram is finally outputted. The schematic diagram of the test process and the preformed speckle pattern of the test piece surface are shown in Figure 3. After the test, the yield strength, ultimate tensile strength and elongation were obtained. In order to observe the special microstructure of the grain boundary, carbides and matrix, a metallographic analysis was carried out by grinding, polishing and electrochemical corrosion of the test alloy. The electrochemical corrosion was carried out in 95 mL HCl (w% ≥ 99.5%) + 10 mL CH3COOH (w% ≥ 99.5%) with a voltage of 4 V and corrosion time of 10 s. The microstructure and microcracks at the fracture surface were observed by optical microscope (OM) (VHX-6000, KEYENCE, Osaka, Japan) and scanning electron microscope (SEM) (ZEISS Gemini300, Carl Zeiss AG, Oberkochen, Germany). The high temperature failure mechanism is explained.

## 3. Experimental Results and Discussion

### 3.1. Tensile Mechanical Behavior at High Temperature

**Figure 4:**
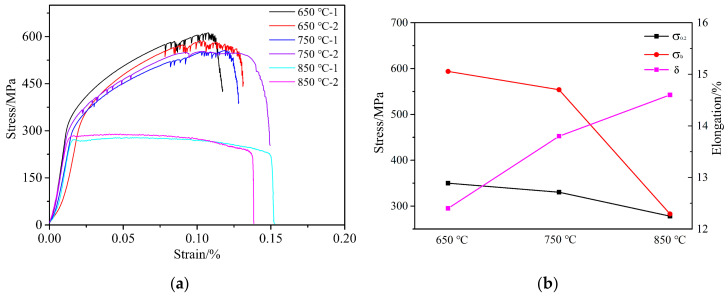
Tensile test results for different temperatures: 650 °C/750 °C/850 °C. (**a**) stress-strain curve of the high-temperature tensile experiment; (**b**) the change trend of high-temperature tensile mechanical properties parameters (σ_0.2_, σ_b_ and δ) with the test temperature.

Since the cross-sectional area of the specimen varies along the tensile direction, the minimum cross-sectional area is used to calculate the stress, and the strain is calculated based on the distance between the two most distant holes along the tensile direction of the specimen. The high-temperature tensile stress-strain curves are shown at different temperatures in Figure 4. The specific high-temperature mechanical parameters of the tested alloy are shown in Table 2. The results show that the specimen has no obvious yield stage under all temperature conditions, so that the stress value corresponding to the 0.2% plastic deformation of the test piece is used as its yield strength (σ_0.2_).

From the stress-strain curve, it can be seen that the mechanical properties of the specimen at 650 °C are the most excellent, with the best values for the yield strength (σ_0.2_) and ultimate strength (σ_b_) but with a lower elongation. The mechanical properties at 750 °C are slightly lower than those at 650 °C, but at 850 °C the yield strength (σ_0.2_) and ultimate strength (σ_b_) of the material are significantly reduced, and there is no strengthening stage. Compared with 650 °C and 750 °C, the yield strength (σ_0.2_) and ultimate strength (σ_b_) at 850 °C are reduced by 20.6% and 52.4%, respectively; however, the elongation (δ) is 1.18 and 1.06 times that at 650 °C and 750 °C, respectively. The variation trends of σ_0.2_, σ_b_ and δ at different temperatures are shown in Figure 4b. It can be inferred that the temperature has no significant effect on the elastic deformation ability of materials. In addition, the stress-strain curves show obvious zigzag fluctuations in the strengthening stage at 650 °C and 750 °C. This phenomenon is usually related to the portevin Le Chatelier effect based on dynamic strain aging (DSA), that is, the pinning effect of diffused solute atoms on dislocations [18,19,20].

The studies in [15,21] found that below 800 °C the yield strength (σ_0.2_) and ultimate tensile strength (σ_b_) of the Ni-Cr-W deformed superalloy decreased slightly as the temperature increased, but that above 800 °C the ultimate tensile strength (σ_b_) dropped drastically, which is consistent with the high-temperature tensile results in our study. Therefore, being the same kinds of material, the micromechanism should be the same. At a high temperature, the main reason for this phenomenon is the existence of the secondary phase M_23_C_6_ at the grain boundary, which reduces the strength of the grain boundary. As a result, cracks initiate at the junction of M_23_C_6_ and the grain boundary, and then propagate, resulting in alloy failure. In addition, the high dislocation density and cross slip of the internal structure of the alloy at a high temperature are also responsible for the phenomenon. Therefore, this kind of material shows a certain sensitivity to temperature. When the temperature is too high, this will lead to the rapid deterioration of its thermal strength.

### 3.2. Mode of Crack Propagation and Strain Field around Holes

**Figure 5:**
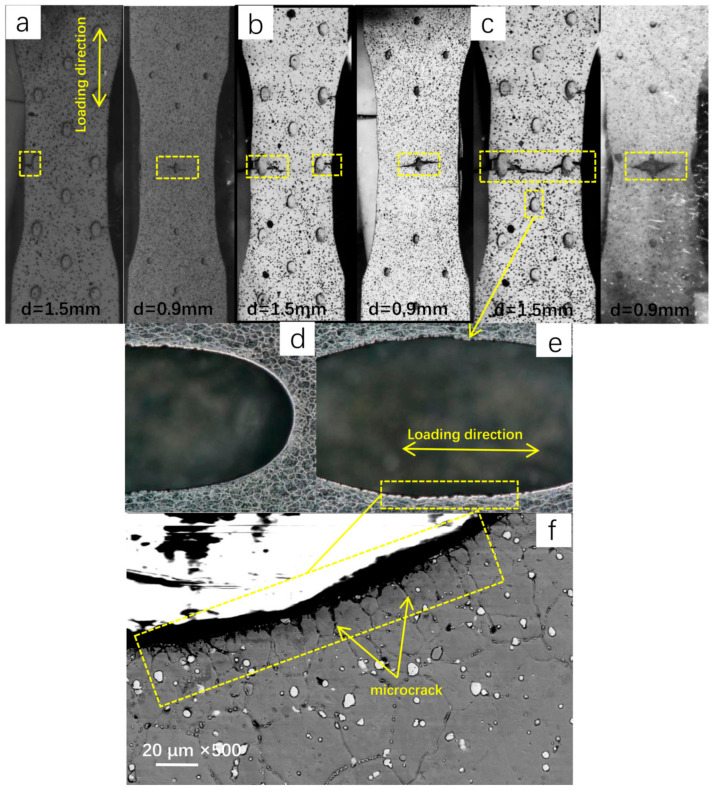
Images of test pieces during testing: (**a**) 650 °C; (**b**) 750 °C; (**c**) 850 °C; (**d**–**f**) microcracks around the hole at 850 °C.

Macroscopic fracture paths are similar at three temperatures. From Figure 5a–c, it can be found that both sides of the test pieces are broken from the middle part of the test piece and that the initial cracks are generated at the middle film-cooling holes. However, due to the differences in the size and arrangement of the impact holes and overflow holes, the crack propagation paths on both sides of the specimen are different. Cracks on the surface of the overflow holes are first generated on the middle overflow hole and expand outward along the direction perpendicular to the loading axis. When the cracks develop toward the edge of the specimen, the edge breaks. The inwardly expanding cracks are in a zigzag shape, which first expands along the direction perpendicular to the load axis, then expands along the 45° direction connecting the two holes, and then returns to the direction perpendicular to the load axis. The cracks on the plate with impact holes are first generated on the middle hole before expanding along the direction perpendicular to the loading axis until they extend to the edge, breaking the entire impact surface. The “zigzag” fracture mode of the plate with overflow holes is mainly caused by the serious stress concentration around the hole at the beginning, resulting in crack initiation. Then, due to the interference effect between the holes, the stress level in the direction 45° to the loading direction is higher, and the crack propagates along the 45° direction. When the crack propagates for a certain distance, the axial stress of the specimen begins to dominate the fracture process, and the crack will propagate along the path that is perpendicular to the loading axis until the plate with overflow holes breaks. Due to the small diameter and sparse layout of the impact holes, the interference effect between the holes is not obvious, causing the cracks to expand along the direction perpendicular to the loading axis until the plate with impact holes fractures. The micromorphology of the overflow hole after the tensile test at 850 °C is shown in Figure 5d–f. It can be seen from the figure that the circular overflow hole has been elongated into an ellipse. Compared with the original test piece, the edge of the overflow hole parallel to the loading direction was complete, but a large number of microcracks were generated at the edge of the hole perpendicular to the direction of the loading axis. Furthermore, the crack initiation position was at the grain boundary. This is mainly due to the machining defects around the pores and the presence of intergranular carbides, which provide a place for microcracks and the nucleation of micropores [13].

**Figure 6:**
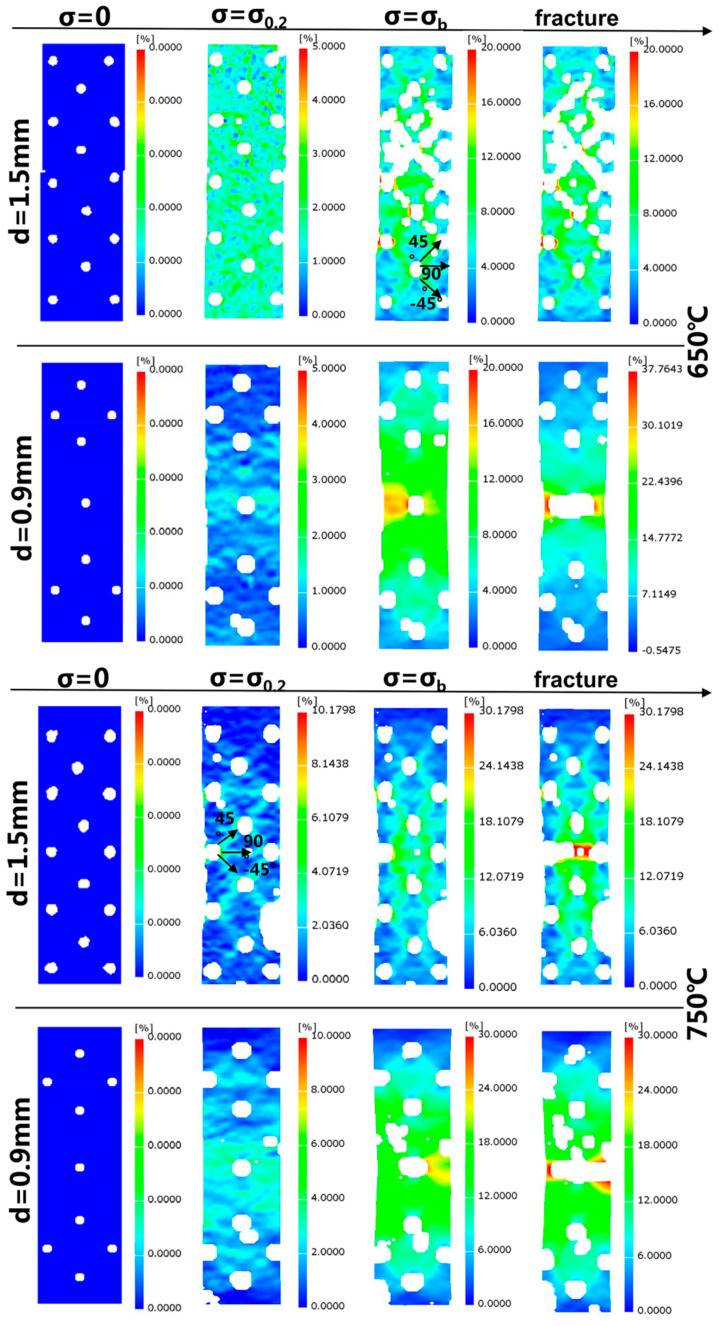

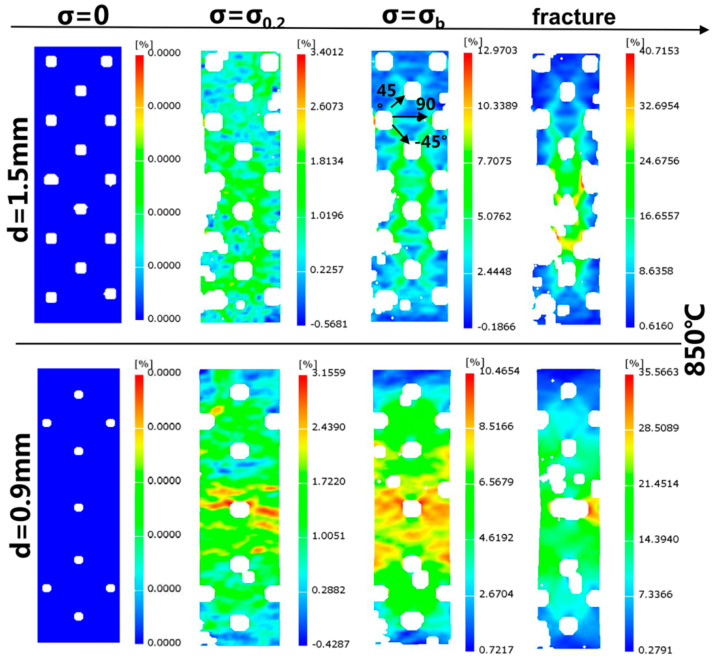
DIC strain nephograms of the specimen plates with overflow holes (d = 1.5 mm) and impact holes (d = 0.9 mm) at three different stress state stages (σ = 0/σ = σ_0.2_/σ = σ_b_) and, before fracture, under three temperature conditions.

The images taken by the high-temperature camera were processed by the DIC system, and the strain nephograms at each temperature were obtained, as shown in Figure 6. Through the strain field nephogram, it can be found that there are obvious hole interference effects between the overflow holes under the three temperature conditions, which shows that there is an obvious high strain band area between the adjacent holes. The results show that the banded zone is 45°, −45° and 90° to the tensile direction and that the maximum strain occurs around the hole where the strain band intersects. It can be seen in Figure 6 that when σ = σ_0.2_, the strain bands are clear. When the ultimate strength is reached, the width of the strain band increases and the high strain area becomes larger. Among these areas, the high strain region between the two big holes in the middle expands the fastest, which makes the middle part of the specimen become a high strain area. Due to the rapid propagation of cracks, the stress drops sharply, but the strain increases slowly. From the strain nephogram, it can also be found that the cracks first occur at the edge of the two holes in the middle before extending to both ends. The propagation of the cracks leads to a sharp increase in the strain in the region between the two holes, which is obviously higher than that in the other regions. When the cracks between the two holes are connected, the plate with overflow holes breaks, and then, 2 to 3 s later, the plate with impact holes fractures, and the whole specimen finally breaks.

By observing the strain nephogram of the plate with impact holes, it is found that the hole interference effect also exists between the impact holes but that it is not as obvious as that between the overflow holes. This is related to the size, density and layout of the impact holes. The strain nephogram has obvious differences at different temperatures. At 750 °C, because the distance between the impact holes arranged in a diamond shape at the upper and lower ends of the specimen is small, strain bands of ±45° and 90° to the tensile direction are formed between the holes when yielding. However, at 650 °C, due to the low temperature and high strength of the material, there is no obvious difference in the strain of each part of the whole face, while at 850 °C the strain of the whole surface is relatively large, and there is also no obvious difference in strain due to the low strength of the material. Because the isolated impact hole is far away from other impact holes, there is no strain band between the isolated hole and other holes under the three temperature conditions. However, the strain around the isolated hole is the greatest. As time increases, the strain in the middle part is obviously higher than for the other parts. The crack propagates from the periphery of the hole along the high strain region to both ends. The fracture mode shown by the strain nephogram is consistent with the actual fracture mode of the specimen in the test.

### 3.3. Fracture Analysis

**Figure 7:**
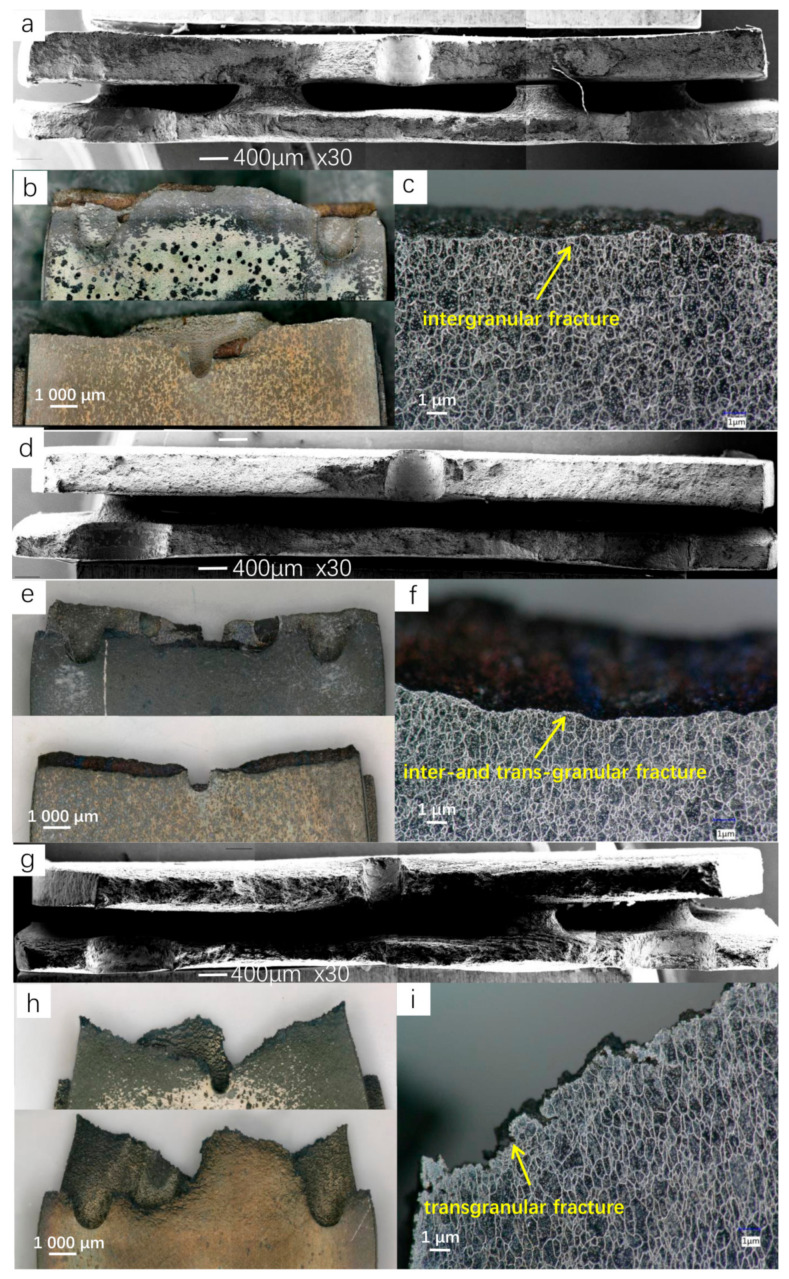
Fracture morphology and side morphology after electrochemical corrosion. (**a**–**c**) 650 °C; (**d**–**f**) 750 °C and (**g**–**i**) 850 °C.

**Figure 8:**
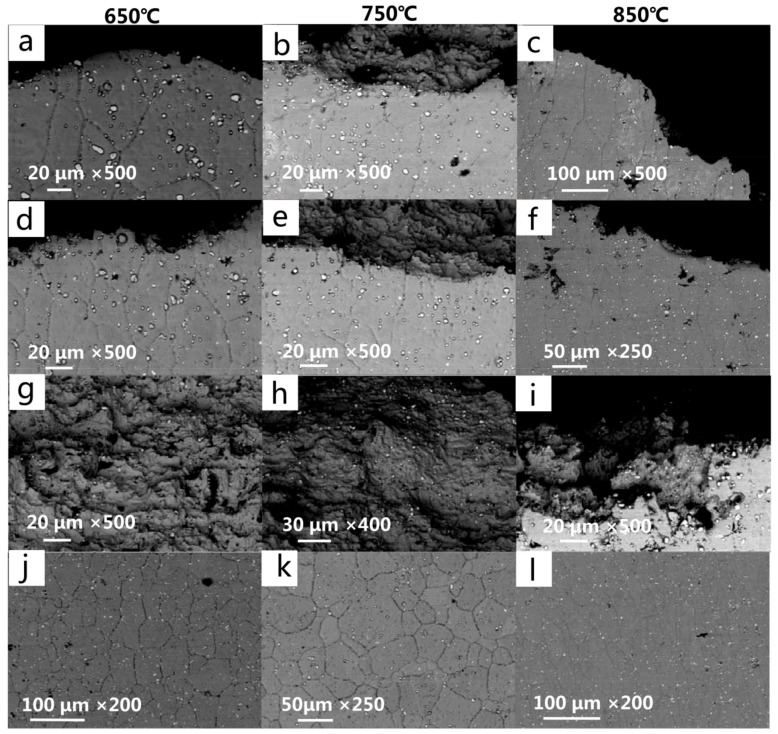
Fracture morphology at 650 °C/750 °C/850 °C. (**a**–**c**) Inclined fracture view of the surface of the plate with overflow holes and (**d**–**f**) the surface of the plate with impact holes; (**g**–**i**) fracture surface morphology and (**j**–**l**) microstructure near the fracture surface.

Figure 7b,e,g shows the fracture morphology seen under the optical microscope under different temperature conditions. It can be seen that the macromorphology corresponds to the fracture mode described in Section 3.2. Under the interference effect between the holes, a crack propagation path appears along the strain band on the plate with overflow holes, while there is no obvious hole interference effect on the plate with impact holes. The fracture morphologies at 650 °C and 750 °C are similar, with the fracture being straight and the Diameter Shrinkage phenomenon not being obvious. However, the fracture surface at 850 °C shows an obvious Diameter Shrinkage phenomenon, and the fracture deformation is serious, which indicates that the plasticity of the material is significantly improved at a high temperature. The fracture surface is observed after electrochemical corrosion, as shown in Figure 7c,f,i, where it can be clearly seen that the fracture at 650 °C is a typical intergranular fracture, the fracture at 750 °C shows the mixed fracture characteristics of an intergranular fracture and transgranular fracture, and the fracture at 850 °C is almost all transgranular fracture. Figure 8 shows the microstructure of the fracture surface and fracture side under SEM. The fracture mode can also be identified by Figure 8a–f, which is consistent with the results observed by optical microscope. In addition, by comparing the microstructure of the fractured test pieces with the microstructure of the material before the test, it can be found that, after the high-temperature tensile test, the amount of carbide on the alloy surface increases when compared with the original structure, but the size decreases slightly. At all temperature conditions, there are no significant differences between the carbides near the fracture surface and those around the holes, but the grains near the fracture surface are obviously elongated. Figure 8g–i shows the micromorphology of the fracture surface after the high-temperature tensile test. It can be seen that all the fractures are ductile dimple fractures, and a large number of second-phase particles are uniformly distributed on the fracture surface. The EDS analysis confirmed that the second-phase particles were granular M_6_C carbides. At the same temperature, the grain size of the plate with overflow holes is larger than that of the plate with impact holes. This is due to the large number and dense arrangement of overflow holes, which makes the stress on the plate with overflow holes larger under the same loading conditions.

### 3.4. High-Temperature Fracture Mechanism of Porous Laminates

The unique structure, which can simulate actual engineering, has a significant influence on the mechanical properties of the test pieces. The effective cross-sectional area is reduced by 73.2–84.6% due to the lamellar and porous structure, resulting in a significant loss of tensile mechanical properties. According to the strain field measured by DIC in Section 3.2, it can be seen that there is an obvious stress concentration area around the holes. Moreover, due to the large diameter and dense arrangement of the overflow holes, there is a significant pore interference effect between the overflow holes under the three temperature conditions. It is shown that there are obvious high strain banded regions between adjacent holes. The direction of the strain band is ±45° and 90° to the tensile direction, and the distribution is symmetrical. The strain around the holes where the deformation bands intersect is the largest, which provides a place for the initiation of cracks on the surface with overflow holes. When the initial microcrack is generated around the holes, the propagation direction of the microcrack is consistent with that of the interference band. When the crack propagates for a certain distance, the effective cross-sectional area decreases, and the stress on the surface with impact holes increases rapidly. The cracks are produced at both ends of the impact hole in the center of the test piece and propagate rapidly to both ends. Then, the cracks on both sides extend outward at the same time until they penetrate the whole test piece, resulting in its fracture failure. The pin fins between the laminates did not bear a direct axial force and did not undergo significant deformation, but they still played a certain role in the strength of the structure itself, which was mainly manifested by bonding the plate with overflow holes and impact holes in order to improve the stiffness of the laminate structure.

**Figure 9:**
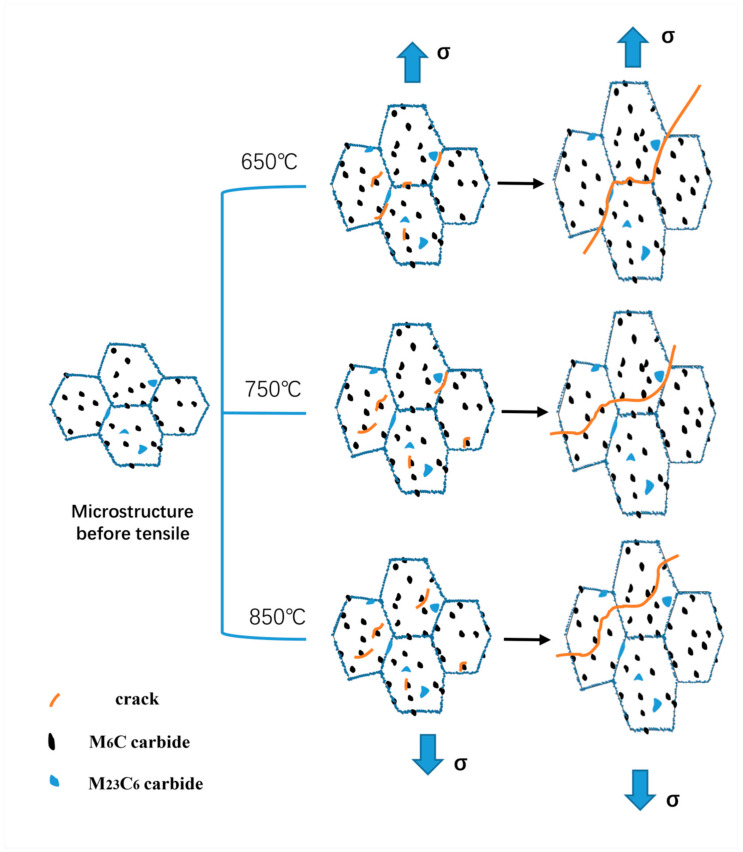
The schematic of the microstructure evolution and fracture mechanism of the tested alloy at different temperatures.

According to the high-temperature tensile stress-strain curve of the alloy in Figure 4, it can be seen that the test alloy shows a significant sensitivity to temperature. The ultimate strength (σ_b_) and yield strength (σ_0.2_) of the specimens are higher at 650 °C and 750 °C but decrease significantly at 850 °C, while the elongation (δ) increases significantly. When the temperature exceeds a certain critical value, the thermal strength of the material will deteriorate rapidly, and the fracture mode of the material will change from intergranular fracture to transgranular fracture with the increase of the test temperature. Under the three temperature conditions, the crack is generated at the grain boundary around the homogeneous hole, as shown in Figure 5f. The reason for this is that the strength of M_23_C_6_ carbide at the grain boundary is high, so that it is difficult for dislocation to cut or bypass it, resulting in the dislocation stacking at the M_23_C_6_-type carbide and forming a dislocation nail. However, when the stress reaches a certain value, the local stress concentration occurs in the γ/M_23_C_6_ bonding area, which makes the cracks appear in the γ/M_23_C_6_ bonding area [20]. From the previous article, one can see that the test material is mainly composed of the matrix phase and two kinds of carbide phases, belonging to the typical carbide dispersion strengthened superalloy. Therefore, the strength of carbides plays an important role in the mechanical properties of the alloy, especially M_23_C_6_ carbide, which can strengthen the grain boundary. At a lower temperature (650 °C), the strength of the matrix phase is greater than that of the grain boundary, so that the crack will propagate along the grain boundary of the interference band between the holes, resulting in an intergranular fracture of the alloy. In addition, after the strengthening stage, the stress-strain curve appears to zigzag due to the cross slip of matrix deformation in the crystal and the pinning effect of diffusion solute atoms on the dislocation motion. With the increase of temperature, both the grain boundary strength and matrix phase strength decrease, but the decrease rate of the matrix strength is faster than that of the grain boundary [22]; when the temperature reaches 850 °C, the strength of the grain boundary will be greater than that of the matrix, and the crack will propagate along the inter-hole strain band. Because the strength of the matrix phase is greatly reduced, the dislocation can easily pass through the M_6_C-type carbide by the climbing mechanism and Orowan bypass mechanism [23]. The crack will propagate through the grain until the specimen breaks. At the same time, due to the decrease of the material strength, the plasticity of the specimen will increase significantly, resulting in a significant increase in the elongation (δ) of the test piece, and the polygonal grains will be elongated into long strips during the tensile test. The microstructure evolution and fracture mechanism of the alloy under high-temperature tensile testing are drawn for different temperatures according to the experimental phenomena, as shown in Figure 9.

## 4. Conclusions

In this study, the Ni-Cr-W superalloy GH3230 was used as the experimental material, and porous laminate specimens were designed in combination with an actual engineering structure. High-temperature tensile tests at 650 °C, 750 °C and 850 °C were carried out. The high temperature mechanical properties and fracture mechanism of deformed superalloy porous laminate specimens were studied by means of digital image correlation (DIC), an ultra depth of field optical microscope, a scanning electron microscope (SEM) and EDS element analysis technology. The main conclusions are as follows:

1. Through the strain field results measured by DIC, it is found that an obvious stress concentration area is formed around the pores of the film-cooling holes, Moreover, due to the large diameter and dense arrangement of the overflow holes, there is a significant pore interference effect between the overflow holes under three temperature conditions. It is shown that there are obvious high strain banded regions between adjacent holes. The direction of the strain band is ±45° and 90° to the tensile direction, and the distribution is symmetrical. On the contrary, no obvious interference effect was found on the side with impact holes, and the crack initiation was mainly due to the stress concentration around the holes. The pin fins between the laminates have no obvious effect on the structural strength, and their function is mainly to bond the laminates on both sides.
2. Because the strength of the matrix phase and carbide phase decreases with the increase of temperature and because the rate of strength reduction differs, the strength of the tested alloy shows a significant temperature sensitivity. The ultimate strength (σ_b_) and yield strength (σ_0.2_) decrease slightly from 650 °C to 750 °C but decrease significantly at 850 °C. The plasticity of the material increases significantly with the increase of temperature. In addition, the stress-strain curves of the tensile tests at 650 °C and 750 °C show characteristic zigzag fluctuations in the strengthening stage due to the pinning effect of the diffusive solute atoms on the dislocation.
3. All the initial microcracks are generated at the grain boundary around the hole perpendicular to the loading direction, before extending macroscopically along the interference band. The interference effect provides a preferential path for the crack growth. On a microlevel, the fracture mode of the specimen changes from intergranular fracture to transgranular fracture with the increase of temperature.

## Figures and Tables

**Table 1:** Chemical composition of the GH3230 alloy (wt.%).

Element	C	Cr	Ni	Co	W	Mo	Al	Ti
Mass fraction/%	0.05–0.15	20.00–24.00	balance	≤5.00	13.00–15.00	1.00–3.00	0.20–0.50	≤0.10
**Element**	**Fe**	**La**	**B**	**Si**	**Mn**	**S**	**P**	**Co**
Mass fraction/%	≤3.00	0.005–0.05	≤0.015	0.25–0.75	0.30–1.00	≤0.015	≤0.05	≤0.50

**Table 2:** Mechanical characteristics of the alloys determined at different testing temperatures.

Temperature	Yield Stress (MPa)	Ultimate Stress (MPa)	Average Yield Stress (σ_0.2_) (MPa)	Average Ultimate Stress (σ_b_) (MPa)	Average Elongation (δ/%)
650 °C	368.99	582.60	350.0	593.8	12.4
	331.08	605.08			
750 °C	332.57	555.16	330.2	553.9	13.8
	327.80	552.68			
850 °C	271.98	277.34	278.0	282.9	14.6
	284.09	288.37			
